# *Drosophila* appear resistant to trans-synaptic tau propagation

**DOI:** 10.1093/braincomms/fcae256

**Published:** 2024-08-08

**Authors:** James H Catterson, Edmond N Mouofo, Inés López De Toledo Soler, Gillian Lean, Stella Dlamini, Phoebe Liddell, Graham Voong, Taxiarchis Katsinelos, Yu-Chun Wang, Nils Schoovaerts, Patrik Verstreken, Tara L Spires-Jones, Claire S Durrant

**Affiliations:** Centre for Discovery Brain Sciences, The University of Edinburgh, Edinburgh EH8 9XD, UK; UK Dementia Research Institute, The University of Edinburgh, Edinburgh EH8 9XD, UK; Centre for Discovery Brain Sciences, The University of Edinburgh, Edinburgh EH8 9XD, UK; UK Dementia Research Institute, The University of Edinburgh, Edinburgh EH8 9XD, UK; Centre for Discovery Brain Sciences, The University of Edinburgh, Edinburgh EH8 9XD, UK; Centre for Discovery Brain Sciences, The University of Edinburgh, Edinburgh EH8 9XD, UK; Centre for Discovery Brain Sciences, The University of Edinburgh, Edinburgh EH8 9XD, UK; Centre for Discovery Brain Sciences, The University of Edinburgh, Edinburgh EH8 9XD, UK; Centre for Discovery Brain Sciences, The University of Edinburgh, Edinburgh EH8 9XD, UK; Schaller Research Group at the University of Heidelberg and the DKFZ, German Cancer Research Center, Proteostasis in Neurodegenerative Disease (B180), INF 581, 69120 Heidelberg, Germany; Faculty of Biosciences, Heidelberg University, INF 234, 69120 Heidelberg, Germany; VIB-KU Leuven Center for Brain & Disease Research, Department of Neurosciences, 3000 Leuven, Belgium; KU Leuven, Department of Neurosciences, Leuven Brain Institute, 3000 Leuven, Belgium; VIB-KU Leuven Center for Brain & Disease Research, Department of Neurosciences, 3000 Leuven, Belgium; KU Leuven, Department of Neurosciences, Leuven Brain Institute, 3000 Leuven, Belgium; VIB-KU Leuven Center for Brain & Disease Research, Department of Neurosciences, 3000 Leuven, Belgium; KU Leuven, Department of Neurosciences, Leuven Brain Institute, 3000 Leuven, Belgium; Centre for Discovery Brain Sciences, The University of Edinburgh, Edinburgh EH8 9XD, UK; UK Dementia Research Institute, The University of Edinburgh, Edinburgh EH8 9XD, UK; Centre for Discovery Brain Sciences, The University of Edinburgh, Edinburgh EH8 9XD, UK; UK Dementia Research Institute, The University of Edinburgh, Edinburgh EH8 9XD, UK

**Keywords:** tau, *Drosophila melanogaster*, trans-synaptic, neurodegeneration, Alzheimer’s disease

## Abstract

Alzheimer’s disease is the most common cause of dementia in the elderly, prompting extensive efforts to pinpoint novel therapeutic targets for effective intervention. Among the hallmark features of Alzheimer’s disease is the development of neurofibrillary tangles comprised of hyperphosphorylated tau protein, whose progressive spread throughout the brain is associated with neuronal death. Trans-synaptic propagation of tau has been observed in mouse models, and indirect evidence for tau spread via synapses has been observed in human Alzheimer’s disease. Halting tau propagation is a promising therapeutic target for Alzheimer’s disease; thus, a scalable model system to screen for modifiers of tau spread would be very useful for the field. To this end, we sought to emulate the trans-synaptic spread of human tau in *Drosophila melanogaster*. Employing the trans-Tango circuit mapping technique, we investigated whether tau spreads between synaptically connected neurons. Immunohistochemistry and confocal imaging were used to look for tau propagation. Examination of hundreds of flies expressing four different human tau constructs in two distinct neuronal populations reveals a robust resistance in *Drosophila* to the trans-synaptic spread of human tau. This resistance persisted in lines with concurrent expression of amyloid-β, in lines with global human tau knock-in to provide a template for human tau in downstream neurons, and with manipulations of temperature. These negative data are important for the field as we establish that *Drosophila* expressing human tau in subsets of neurons are unlikely to be useful to perform screens to find mechanisms to reduce the trans-synaptic spread of tau. The inherent resistance observed in *Drosophila* may serve as a valuable clue, offering insights into strategies for impeding tau spread in future studies.

## Teaser

Catterson *et al.* report that *Drosophila melanogaster* does not emulate the trans-synaptic spread of human tau, making it unsuitable for screening anti-tau propagation therapeutics. This inherent resistance may provide insights for future studies aiming to impede tau spread in Alzheimer’s disease.

## Graphical Abstract

**Graphical Abstract:**
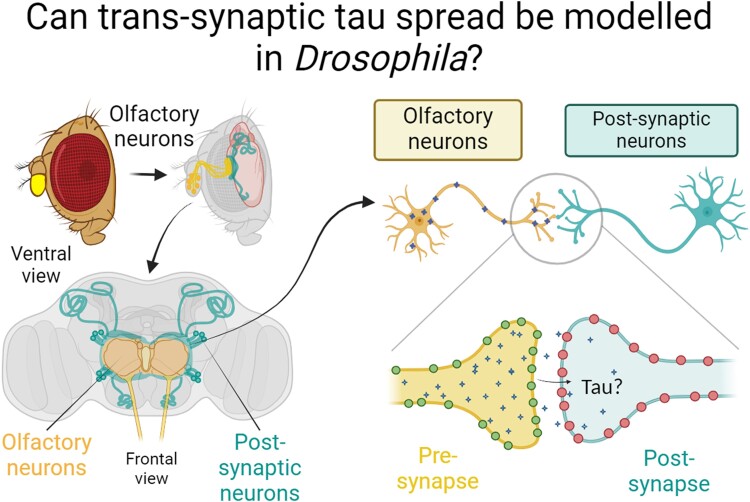

## Introduction

Accumulation of tau pathology in Alzheimer’s disease and other tauopathies is associated with neuron death, synapse loss and decline in brain function.^1^ In Alzheimer’s disease, pathological tau appears early in the disease process in the entorhinal cortex and brainstem and then progresses to synaptically connected brain regions in such a stereotypical fashion that the pattern of tau pathology is used to determine the stage of disease in post-mortem tissue.^2^ This progressive tau spread has also been confirmed longitudinally in living people with Alzheimer’s disease through PET with tracers that bind tau pathology.^3-5^ Similarly, in primary tauopathies, tau pathology generally starts in restricted brain regions and spreads through the brain. For example in progressive supranuclear palsy, post-mortem investigations indicate that tau pathology accumulates early in the brainstem, midbrain and basal ganglia and spreads to the cerebellum and neocortex later in the disease.^6^

There is converging evidence suggesting that pathological tau spreads between brain regions via synapses by neurons from one brain region releasing tau from pre-synapses that is then taken up by post-synapses in neurons in the downstream brain region. In mice, expression of human tau (hTau) restricted to entorhinal cortex, either by viral injection or use of the neuropsin promotor to drive a transgene, causes both tau accumulation in the entorhinal cortex and the accumulation of tau in downstream brain regions in the hippocampus.^7-12^ In mice expressing both hTau and pre-synaptic vesicles tagged with green fluorescent protein (GFP), we detected hTau in post-synapses in dentate gyrus opposed to hTau-expressing pre-synapses from entorhinal cortex, directly demonstrating trans-synaptic tau spread in this model.^11^ Injecting tau derived from post-mortem brain tissue from people who died with tauopathies also induces tau aggregation in mouse models, which spreads from the injection site to connected brain regions.^13-16^ While these mouse systems have confounds which could aberrantly drive trans-synaptic spread, there is also mounting human evidence supporting this mechanism of tau propagation. In Alzheimer’s disease and primary tauopathies progressive supranuclear palsy and cortico-basal degeneration, MRI studies indicate that tau accumulates in functionally connected brain regions.^17-19^ Using high-resolution array tomography imaging, we observe phosphorylated tau within synapses^11,20^ and even find pathological tau within synaptic pairs in brain regions affected late in the disease,^21,22^ supporting trans-synaptic spread of tau.

Wherever tau pathology appears in the brain, neurons and synapses die, making preventing tau spread a promising therapeutic target. While mouse model systems robustly exhibit trans-synaptic tau spread, they can take many months to develop a phenotype and are costly and time-consuming models to use for identification of modifiers of tau spread. Human-induced pluripotent stem cell-derived neurons have also been used to model tau spread,^23^ providing a human-relevant *in vitro* model. These cells, however, express largely embryonic forms of tau,^24^ which may not be representative of adult physiological functions,^25^ and lack intact brain circuits, limiting their utility. *Drosophila melanogaster* has the advantages of short lifespan and a range of molecular tools developed to manipulate gene expression in various cell types. *Drosophila* have been used to study the effects of pathological tau to great effect. hTau expression in *Drosophila* has been shown in many studies with different tau transgenes and drivers to induce neurodegeneration and has been used to great utility in identifying modifiers of tau toxicity.^26-30^ Of particular interest, tau in *Drosophila* has been implicated in neurodegeneration through binding pre-synaptic vesicle proteins synaptogyrin-3 and bassoon^31-33^ and synaptic pathways modulate tau toxicity in flies.^30^ Tau expressed in the developing *Drosophila* retina has been observed to spread out of the retina into the optic lobe^34-36^; however, it was unclear in these studies whether this was trans-synaptic propagation between neurons or whether the observed signal was due to degenerating axon terminals from the retina or glia taking up secreted tau.

In this study, we have harnessed the trans-Tango system in *Drosophila*^37^ to express hTau and GFP in restricted neuronal populations, and tdTomato in synaptically connected downstream neurons, to allow us to address whether tau spreads trans-synaptically in this model system. We further test the influence of several factors that modulate tau spread in mammals in this *Drosophila* system. Amyloid-β (Aβ) pathology has been shown to exacerbate trans-synaptic tau spread in mouse models,^38^ and recent data in humans support that synaptic effects of Aβ induce tau accumulation^39^; thus, we examine potential tau spread in the context of concurrent human Aβ expression. Although in mice the knock-out of mouse tau did not exacerbate hTau spreading phenotypes,^40^ we hypothesized that detection of tau spreading in flies could be enhanced if low levels of hTau were present in downstream neurons to allow templated misfolding of the tau spreading from overexpressing neurons. In mice, older animals exhibit more trans-synaptic tau spread^10^ and tau propagation out of the retina was exacerbated by age in *Drosophila*^34,36^; thus, we also examined the effect of aging in our novel system by examining flies up to 4–6 weeks of age.

## Materials and methods

### Fly husbandry and stocks

Unless otherwise stated, flies were maintained and all experiments were conducted at 25°C on a 12-h:12-h light/dark circadian cycle using standard sugar/yeast/agar medium.^41^ Only female flies were analysed for the current study. Unless otherwise stated, ‘control’ genotype groups are conditions that use a ‘without hTau’ control cross (typically w^Dah^ or UAS-mCD8:GFP).

The following stocks were obtained from the Bloomington *Drosophila* Stock Center: PDF-GAL4 (#6900, now #80939), Orco-GAL4 (#26818), GH146-QF, QUAS-mCD8:RFP (#30037), UAS-mCD8:GFP (#5137), UAS-mCD8:RFP (#27392), UAS-myr:GFP, QUAS-mtdTom:HA, trans-Tango (#77124), QUAS-Aβ_40_ (#83346), QUAS-Aβ_42_ (#83346), UAS-hTau^WT(0N4R)^ (#78847), and UAS-hTau^WT(2N4R)^ (#78861). The control w^Dah^ was a kind gift from Dr Linda Partridge (UCL, UK).^42^ UAS-hTau^P301L(2N4R)^ was a kind gift from Dr Bess Frost (UT Health San Antonio, USA).^43^

### Trans-Tango

The trans-Tango system in *Drosophila* (see Supplementary Fig. 1A) utilizes a synthetic signalling pathway to map neural circuits.^37^ All neurons express a trans-Tango glucagon receptor (G protein coupled) and a proteolytic complex that can cleave the trans-Tango receptor (Arr-TEV). Specific pre-synaptic neurons are genetically labelled using the GAL4/UAS system to drive expression of a tethered ligand (membrane-bound synaptic protein with tethered glucagon) and genes of interest (e.g. GFP, hTau). This ligand binds to the trans-Tango receptor on post-synaptic partners, triggering release of a transcription factor (QF) from the receptor via proteolysis. The reporter translocates to the nucleus where it binds QUAS elements allowing labelling of post-synaptic neurons (e.g. tdTom).

### Generation of hTau transgenic flies

#### Generation of UAS-hTau^0N4R(WT)^:HA and UAS-hTau^0N4R(E14)^:HA flies

For generation of the C-terminally 3xHA-tagged tau transgenic flies, the coding sequence of hTau isoform 0N4R (UniProt ID: P10636-6) was subcloned into the pJFRC7 vector (Addgene, plasmid #26220, http://n2t.net/addgene:26220; RRID:Addgene_26220, a gift from Gerald Rubin^44^) to allow expression under the control of the GAL4/UAS system. As well as the wild-type version, a phosphomimetic variant of tau (E14) was generated, where 14 serine and threonine residues were mutated to glutamate residues to mimic hyperphosphorylation.^45^ The transgenic flies were generated by phiC31 integrase-mediated transgenesis^46^ using attP landing sites 25C6 on the second chromosome and 68A4 on the third chromosome. Plasmid injections and generation of the 3xHA-tagged hTau fly lines were performed by the Cambridge Department of Genetics Fly Facility. To achieve precise integration of the hTau gene, we utilized phiC31 integrase-mediated transgenesis.^47^ This method leverages phiC31 integrase to insert the gene construct (including the hTau sequence with a 3xHA tag) into predetermined locations within the fly’s genome. Unlike random integration methods, this approach minimizes disruption of essential genes and offers consistent gene expression levels. White-eyed transgenic flies containing the appropriate landing site and a phiC31 integrase transgene (under germline promoter expression) are injected with a plasmid carrying the hTau gene and a visible marker. In this case, the marker is a gene for a distinct eye colour, such as red. Following injection into very early-stage embryos, the flies are allowed to develop. By screening for red eye colour in subsequent generations, researchers can efficiently identify flies that carry the hTau transgene.

#### Generation of hTau knock-in flies

hTau knock-in flies were generated using the CRISPR/Cas9 technique employing a two-step method. Initially, a donor plasmid (pWhite-star) and a tandem gRNA-expressing plasmid (pU6-gRNA) were utilized to generate a *Drosophila* tau (dTau) knock-out. The first exon of dTau was replaced with a mini-white expressing cassette through homology-directed repair. Subsequently, point mutations of hTau cDNA were introduced using PhiC31 integrase-mediated cassette exchange with pKI plasmids.

#### Generation of vectors for dTau knock-out

Two distinct gRNA sequences targeting dTau were identified:

- gRNA1_Tau: TCAAACGTATGGTCTCTGCA
- gRNA2_Tau: CACTTTTACTTACTAAATTC

These gRNA sequences were introduced into the pU6-BbsI-chiRNA plasmid by restriction/ligation into the BbsI site. The resulting gRNA-expressing plasmid was named pU6-gRNA-Tau. pU6-BbsI-chiRNA was obtained from Addgene (plasmid #45946; http://n2t.net/addgene:45946; RRID: Addgene_45946, a gift from Melissa Harrison, Kate O'Connor-Giles and Jill Wildonger).^48^

Homology arms of 1 kb surrounding the first exon of dTau were polymerase chain reaction (PCR) amplified from *Drosophila* genomic DNA using the following primers:

- F_HA1_Tau: GCCACTAGTAGAGACCATACGTTTGACATTCGGG
- R_HA1_Tau: CAGCTCGAGGGCGTTCGGTTCGGGTGT
- F_HA2_Tau: CAGCTCGAGGGCGTTCGGTTCGGGTGT
- R_HA2_Tau: CAGGGTACCGCTACAGCAGCGGAGCATTG

This PCR generated two DNA fragments with SpeI–XhoI and KpnI–AvrII restriction sites, which were used for cloning the fragments into the pWhite-STAR plasmid^49^ via restriction/ligation. The resulting plasmid was named pWhite-STAR_HA-Tau.

#### Generation of vectors for hTau knock-in

To create the hTau knock-in plasmids, a new backbone containing the 3′ and 5′ UTRs of dTau, with a XhoI restriction site in between for integration of hTau cDNA, was generated. Two fragments were PCR amplified from *Drosophila* genomic DNA using the following primers:

- F_UTR_frag1: CTCACCCATCTGGTCCATCATGATGCTAAGTGCAACAACGCCGAGA
- R_UTR_frag1: CAGCTCGACATGCATTTCGGCATCTCGAGATTGAAAGTCGA
- F_UTR_frag2: GCATTTCGGCATCTCGAGATTGAAAGTCGAACGAGTGTGTGTG
- R_UTR_frag2: CTCACCCATCTGGTCCATCATGATGGCACGGTGATTGCGTCTTG

These fragments were cloned into the MiMIC 1322 pBS-KS-attB1-2 plasmid^50^ after digestion with XbaI and EcoRI, using Gibson assembly. The resulting plasmid was named pKI_UTR_Tau. Next, hTau^WT(0N4R)^ and hTau^WT(2N4R)^ cDNAs were PCR amplified with the following primers:

- F_cDNA_Tau: GACCTCGAGATGGCTGAGCCCCGCCAG
- R_cDNA_Tau: GACCTCGAGTCACAAACCCTGCTTGGCCAG

These PCR products were then cloned into the XhoI restriction site of the pKI_UTR_Tau plasmid via restriction/ligation, resulting in the plasmids pKI_Tau^WT(0N4R)^ and pKI_Tau^WT(2N4R)^. All plasmids were sequence verified, and knock-out and knock-in plasmid injections were performed by BestGene (BestGene Inc., Chino Hills, CA, USA).

### Brain dissections and immunostaining

Using forceps, fly heads were removed from the bodies in cold phosphate buffered saline (PBS) containing 4% paraformaldehyde (PFA, Pierce, Thermo Fisher) + 0.01% Triton X-100 (Sigma). The proboscis was then removed from the fly head to allow fixative and detergent to fix and permeabilize the fly brain. Fly heads were transferred to a 0.6 mL microcentrifuge tube (Fisherbrand™ Premium Microcentrifuge Tubes), fixed in 400 μL 4% PFA + 0.01% Triton X-100 and rocked for 16 min at room temperature. Heads were washed with PBST-1 (PBS containing 0.01% Triton X-100) for 3 × 2 min at room temperature. Brains were dissected from the fly heads in ice-cold PBST-1 in a dissection dish under a light stereomicroscope. Brains were transferred to a new 0.6 mL microcentrifuge tube, fixed in 400 μL 4% PFA + 0.1% Triton X-100 and rocked for 20 min at room temperature. Brains were then washed in PBST-2 (PBS containing 0.1% Triton X-100) for 3 × 2 min and blocked with SeaBlock blocking buffer (Thermo Fisher) for 15 min at room temperature. SeaBlock blocking buffer was removed, and brains were incubated in primary antibody in PBST-2 overnight, washed in PBST-2 for 3 × 20 min and incubated in secondary antibody in PBST-2 for 1 h in the dark. Brains were washed in PBST-2 for 3 × 20 min at room temperature in the dark and mounted on a microscope slide using Vectashield mounting media (Vector Labs). To maintain the 3D structure of the brain, a bridge was made during mounting consisting of two round cover slips (VWR, 13 mm, thickness 0) which meant the brains were not crushed.

The following antibodies were used: primary antibodies [final concentrations (if known) are listed]—Mouse anti-Alz50 (1:250, #Alz50 serum, courtesy of Dr Peter Davies, Albert Einstein College of Medicine, USA), Mouse anti-AT180 [1:250 (0.8 μg/mL), #MN1040, Thermo Fisher], Mouse anti-AT8 [1:250 (0.8 μg/mL), #MN1020, Thermo Fisher], Chicken anti-GFP [1:1000 (10 μg/mL), #GFP-1020, Aves Labs], Rat anti-HA [1:1000 (0.1 μg/mL), #11867423001, Roche], Rat anti-NCAD [1:20 (1.35 μg/mL), #DN-Ex8, Developmental Studies Hybridoma Bank (DSHB)], Rabbit anti-RFP [1:250 (2 μg/mL), #ab62341, Abcam], Rabbit anti-T22 (1:250, #T22 serum, courtesy of Dr Rakez Kayed, University of Texas Medical Branch, USA), Goat anti-Tau [aka Tau Goat, 1:250 (0.8 μg/mL), #AF3494, R&D Systems], Mouse anti-Tau13 [1:250 (4–12 μg/mL), #835201, BioLegend], and Mouse anti-Tau 5A6 [1:50 (1.26 μg/mL), #5A6, DSHB].

Secondary antibodies (all 1:400) were as follows: Donkey anti-Chicken 488 IgG (#A78948, Thermo Fisher), Donkey anti-Goat IgG 647 (#A21447, Thermo Fisher), Donkey anti-Mouse 488 IgG (#A21202, Thermo Fisher), Donkey anti-Mouse 647 IgM (#ab150123, Abcam), Donkey anti-Mouse 647 IgG (#ab150107, Abcam), Goat anti-Rabbit 546 IgG (#A11035, Thermo Fisher), Donkey anti-Rabbit 647 IgG (#A32795, Thermo Fisher), Donkey anti-Rat 488 IgG (#A21208, Thermo Fisher), Donkey anti-Rat 555 IgG (#A21434, Thermo Fisher), and Donkey anti-Rat 647 IgG (#A21247, Thermo Fisher).

### Microscopy and image analysis

Images were captured with a Leica TCS SP8 confocal microscope (Leica, Wetzlar, Germany) with a ×63 oil immersion objective. Images were taken as stacks and are shown as single sections or maximum intensity projections of the stack. All images for one experiment were taken at the same settings. All images were analysed and processed using ImageJ (https://imagej.net/). Brain imaging was performed one brain per genotype, minimizing potential order effects within each genotype. This ensured that all brains within a specific genotype were treated and measured in the same order, preventing any systematic bias related to the order of processing.

We used the ‘Blind Analysis Tools’ ImageJ plug-in (https://imagej.net/plugins/blind-analysis-tools) to blind files before scoring or quantification. Images were ‘yes/no’ scored for the presence of hTau outside expressing neurons, for the presence of hTau inside post-synaptic neurons or for the presence of AT8 in specific subsets of neurons. To measure brain area, we used the ImageJ polygon tool to outline and then measure the part of the brain with the largest area (typically the last slice of the confocal stack). For fluorescence intensity, images were separated into individual channels, thresholded, and then the relevant channel was stacked using Z-stacks with the ‘Sum Slices’ function and then measured to get mean and maximum intensity values. For signal co-localization, we thresholded and segmented individual channels then used the ‘Image Calculator’ function to subtract the pigment dispersal factor (PDF) signal from the hTau signal. This resulting image was multiplied by the tdTom:HA signal resulting in a new channel representing hTau signal outside PDF neurons and inside post-synaptic neurons. This was then stacked using Z-stacks with the ‘Sum Slices’ function and then measured to get mean and maximum intensity values.

### Study design

Sample sizes for this study were determined by practical limitations and established practice. The number of brains that could be dissected in a single morning was a primary consideration when taking into account potential circadian effects. Sample sizes align with the standard sample size typically used in similar studies within this field. Each data point represents an individual fly brain, and no data points were excluded. Each figure legend that contains quantified data has all of the *n* numbers listed. Randomization was not employed in allocating experimental units to control and treatment groups. While acknowledging the importance of randomization for reducing bias, it was not feasible in this specific study due to the logistical difficulty of randomizing fly brains on microscope slides. However, to mitigate potential bias, we implemented blinding during the experiments. For the experiment assessing the effect of post-synaptic Aβ, microscope slides were blinded by C.S.D. before J.H.C. conducted imaging. In all other instances, the researcher was not fully blinded from the group allocation throughout the experiment. However, as previously mentioned, blinding was performed on all acquired images to be analysed or scored.

### Data presentation and statistical analysis

Analysis and visualization of the data were facilitated using the ‘ggplot2’ package in R (RRID:SCR_001905) and RStudio (RRID:SCR_000432, https://posit.co/). The large language models ChatGPT (OpenAI) and Gemini (Google) were employed to assist in refining R code. Data were grouped for each genotype and were displayed as boxplots or stacked bar charts using the ‘ggplot2’, ‘ggbeeswarm’, and ‘ggpattern’ packages. Cartoons were generated using BioRender (RRID:SCR_018361, http://biorender.com). Inkscape (RRID:SCR_014479, http://www.inkscape.org/) was used to generate figures.

Statistical analysis was performed using R and RStudio. Statistical tests used include one-way ANOVA, Kruskal–Wallis rank sum test, Wilcoxon rank sum test, *χ*^2^ and Fisher’s exact test, and the appropriate *post hoc* analyses were performed. If the model did not meet the assumptions (assessed by plotting histograms to check for normality, plotting model residuals against predictors, normality of residuals was checked with a QQ plot, and homogeneity of variance was checked by plotting residuals against fitted values), data were transformed using the method that best transformed each individual model to fit the assumptions, for example Tukey transformation. Statistical details for each individual analysis can be found in the ‘Results’ text. Significance values were reported as ***P < 0.001.

## Results

### Wild-type and phosphomimetic hTau stays restricted to expressing neurons, even in flies kept at 29°C for 4 weeks

**Figure 1:**
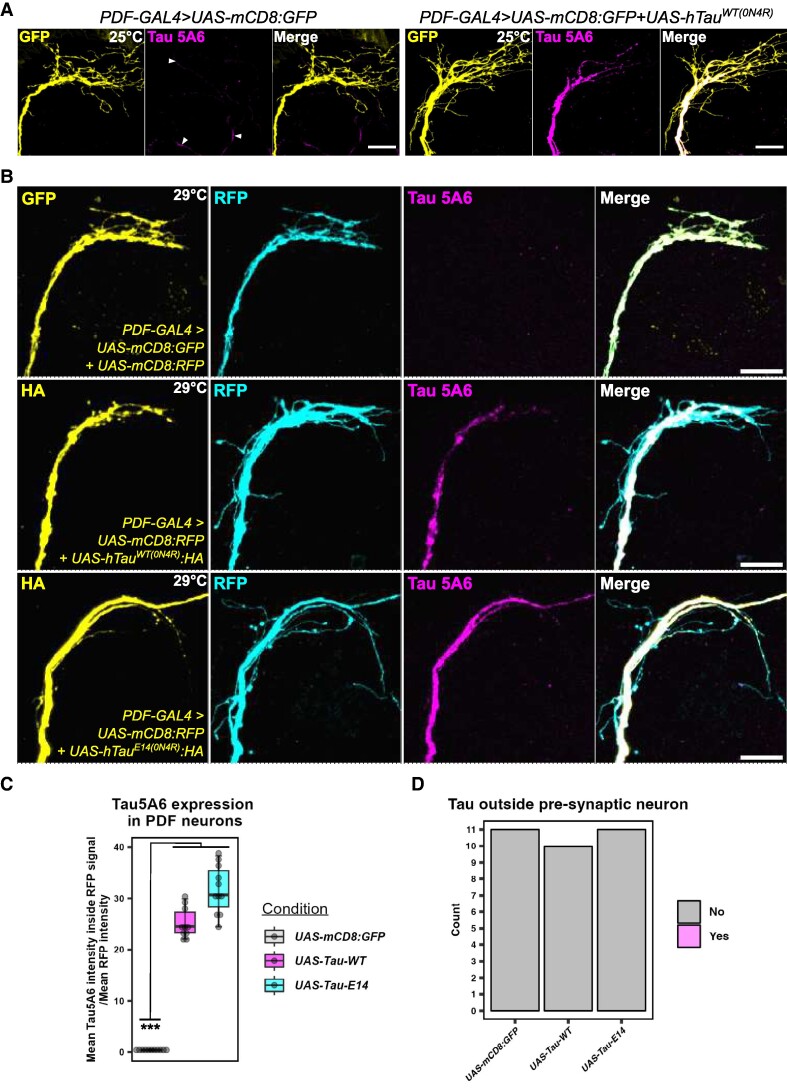
**hTau stays restricted to expressing neurons, even in flies kept at 29°C for 4 weeks.** (**A**) hTau staining was restricted to within the PDF neuron membrane—marked by membrane-bound GFP (mCD8:GFP). Control flies without hTau exhibited no Tau 5A6 staining, unlike Tau13-stained controls in Supplementary Fig. 1. (**B**) In flies kept at 29°C for 4 weeks, Tau 5A6 staining remained restricted to within the PDF neuron membrane—marked by membrane-bound red fluorescent protein (mCD8:RFP). Control flies without hTau that expressed both GFP and RFP [to control for two upstream activation sequence (UAS) lines] exhibited no Tau 5A6 staining. Haemagglutinin (HA) was also imaged and was found to overlap with RFP. (**C**) Fluorescence intensity of co-localized Tau 5A6-RFP was measured and normalized to the mean RFP intensity of each brain. Tau 5A6 detected significant hTau overexpression in PDF neurons compared to GFP controls (Kruskal–Wallis rank sum test, *χ*^2^ = 25.2, df = 2, *P* < 0.0001; *N* = 10–11 brains). There was no significant difference between hTau^WT^ and hTau^E14^ expression (*P* = 0.12, Dunn *post hoc* test, *P*-value adjusted with the Bonferroni method). (**D**) Tau 5A6 staining outside RFP-labelled PDF neurons was not observed. Images were blinded and scored, and no difference between genotypes was observed (*χ*^2^ test, *χ*^2^ = 0.063, df = 2, *P* = 0.97; *N* = 10–11 brains). Scale bar is 20 μm. Genotypes: (**A**) *PDF-GAL4 > UAS-mCD8:GFP*, *PDF-GAL4 > UAS-mCD8:GFP + UAS-hTau^WT(0N4R)^*. (**B**) *PDF-GAL4 > UAS-mCD8:GFP + UAS-mCD8:RFP*, *PDF-GAL4 > UAS-mCD8:RFP + UAS-hTau^WT(0N4R)^:HA*, *PDF-GAL4 > UAS-mCD8:RFP + UAS-hTau^E^*^14*(0N4R)*^*:HA*.

We chose to overexpress hTau in membrane-bound fluorophore-labelled PDF neurons using the binary GAL4/UAS system and then looked to see if we could observe hTau outside of the expressing neurons. We chose PDF neurons because they are a distinct, well-characterized set of neurons (∼16 total)^51^ that also have axon termini located distally from their cell bodies. We focused on the dorsal axon termini adjacent to the mushroom body calyx as this region is abundant with PDF neuron pre-synapses.^52^ In our initial attempts to characterize hTau spread in *Drosophila*, we decided to use the monoclonal hTau antibody commonly used in mammalian tau spread studies^38,53,54^—Tau13 (Supplementary Fig. 1B). However, we observed non-specific labelling of structures in post-synaptic neurons and in the mushroom body calyx in controls without hTau overexpression that were not present in no-primary antibody control conditions (Supplementary Fig. 1C). We observed a similar non-specificity with the Tau Goat antibody with this antibody detecting apparent cell bodies (Supplementary Fig. 1D and E). Thus, we tested other commonly used hTau antibodies (Tau 5A6, AT8, AT180, T22 and Alz50) in flies expressing two copies of C-terminal 3xHA-tagged hTau and found that all could label hTau in PDF neurons, though the signal for T22 and Alz50 was reduced likely due to lower levels of oligomeric or misfolded hTau species compared to monomeric forms (Supplementary Fig. 2). We decided to use the monoclonal Tau 5A6 antibody to label hTau as it appeared specific and could detect full-length hTau. We confirmed hTau expression in PDF neurons and observed hTau was highly restricted to PDF neurons with no obvious tau spread outside the membrane–GFP signal (Fig. 1A). Since we did not observe hTau outside of expressing neurons, we hypothesized that increasing temperature could force tau propagation as raising hTau-expressing flies at higher temperatures is known to exacerbate tau toxicity.^55^ We generated flies that expressed UAS-mCD8:RFP (to label pre-synaptic neurons) and wild-type UAS-hTau^WT(0N4R)^ or toxic, phosphomimetic UAS-hTau^E14(0N4R)^ (both C-terminally 3xHA-tagged) in PDF neurons and aged flies for 4 weeks. To control for multiple UAS lines, we used a control that expressed both UAS-mCD8:GFP and UAS-mCD8:RFP (Fig. 1B). We quantified fluorescence intensity of hTau in each genotype and found an expected significant hTau overexpression in PDF neurons compared to GFP controls (Kruskal–Wallis rank sum test, *χ*^2^ = 25.2, df = 2, *P* < 0.0001) (Fig. 1C). There was no significant difference between hTau^WT^ and hTau^E14^ expression (*P* = 0.12, Dunn *post hoc* test, *P*-value adjusted with the Bonferroni method). However, hTau staining outside RFP-labelled PDF neurons was not observed (*χ*^2^ test, *χ*^2^ = 0.063, df = 2, *P* = 0.97) (Fig. 1D).

### Absence of hTau propagation to post-synaptic neurons with different hTau isoforms and different post-synaptic Aβ isoforms

**Figure 2:**
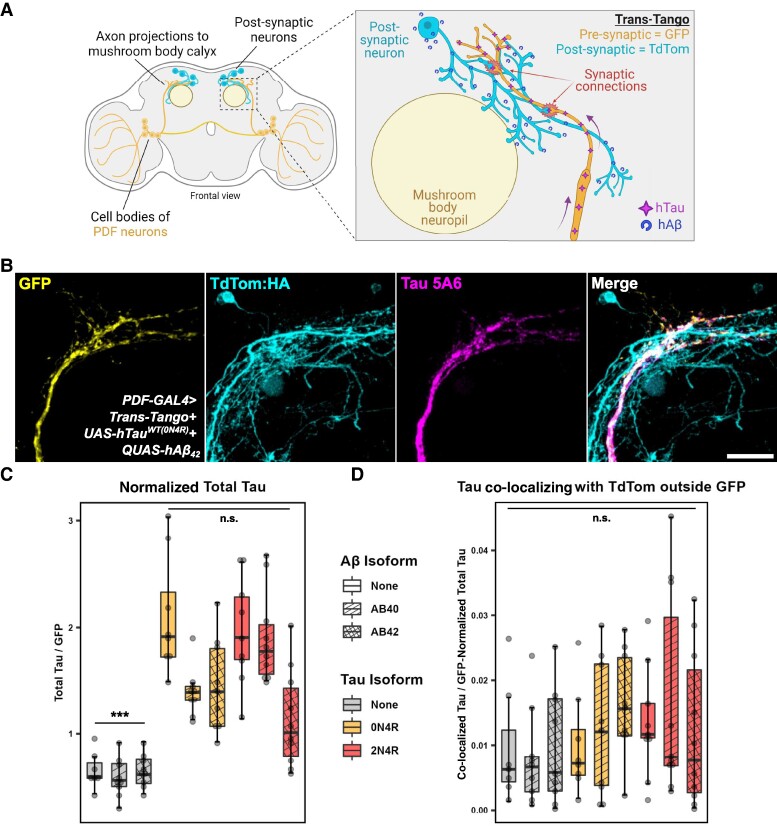
**Absence of hTau propagation to post-synaptic neurons with different hTau isoforms and different post-synaptic Aβ isoforms.** (**A**) PDF neurons project to the mushroom body calyces. Trans-Tango labels pre-synaptic PDF neurons with GFP and their post-synaptic partners with haemagglutinin (HA)-tagged tdTomato (tdTom). Additional experiments using GAL4 and QF drivers (from the trans-Tango system) can also express hTau and human Aβ (hAβ) in pre- and post-synaptic compartments. Created using BioRender. (**B**) Representative axon terminal region from PDF neurons highlighting GFP and hTau expression in PDF neurons, and tdTom:HA expression in post-synaptic partners. This fly also expressed Aβ_42_ in post-synaptic neurons. Staining with Tau 5A6 antibody (full-length hTau) indicated hTau expression in the PDF neuron but not outside. Scale bar = 20 μm. (**C**) Quantification of total hTau fluorescence intensity showed an expected significant increase in hTau-expressing brains compared to controls without hTau (this difference is indicated by *** above the conditions without hTau; main effect of hTau isoform: one-way ANOVA on Tukey-transformed data: *F*(2,77) = 58.73, *P* < 0.0001; *N* = 7–10 brains). (**D**) Quantification of hTau co-localization signal outside GFP and inside tdTom signals showed no difference between genotypes (main effect of genotype: one-way ANOVA on Tukey-transformed data: *F*(8,71) = 0.78, *P* = 0.62; *N* = 7–10 brains). n.s. = not significant. Genotypes (left to right): *PDF-GAL4 > trans-Tango*; *PDF-GAL4 > trans-Tango + QUAS-Aβ_40_*; *PDF-GAL4 > trans-Tango + QUAS-Aβ_42_*; *PDF-GAL4 > trans-Tango + UAS-hTau^WT(0N4R)^*; *PDF-GAL4 > trans-Tango + UAS-hTau^WT(0N4R)^+QUAS-Aβ_40_*; *PDF-GAL4 > trans-Tango + UAS-hTau^WT(0N4R)^+QUAS-Aβ_42_*; *PDF-GAL4 > trans-Tango + UAS-hTau^WT(2N4R)^*; *PDF-GAL4 > trans-Tango + UAS-hTau^WT(2N4R)^+QUAS-Aβ_40_*; *PDF-GAL4 > trans-Tango + UAS-hTau^WT(2N4R)^+QUAS-Aβ_42_*.

Since we could not detect hTau spreading outside of expressing neurons, we hypothesized that a second insult may need to occur to facilitate trans-synaptic hTau spread. We took advantage of the anterograde trans-synaptic tracing tool trans-Tango^37^ to monitor the potential spread of ectopically expressed hTau in PDF neurons (Fig. 2A and B). The trans-Tango system labels pre- and post-synaptic neurons with different membrane-tagged fluorophores—GFP and tdTom, respectively (see Supplementary Fig. 1A for schematic). We leveraged the ability of the trans-Tango system’s use of QF expression in post-synaptic neurons to express either QUAS-Aβ_40_ or QUAS-Aβ_42_ as well as QUAS-tdTom (Fig. 2A) while expressing UAS-mCD8:GFP and either UAS-hTau^WT(0N4R)^ or UAS-hTau^(2N4R)^ isoforms in pre-synaptic neurons (representative example image in Fig. 2B). This approach minimizes potential interactions between Aβ and the hTau expression machinery within the same neuron. By keeping them separate, we can more clearly observe how extracellular Aβ might influence the physiology of neurons expressing hTau. We quantified total hTau fluorescence and found a significant increase among all hTau-expressing brains regardless of hTau isoform compared to controls without hTau expression (main effect of hTau isoform: one-way ANOVA on Tukey-transformed data: *F*(2,77) = 58.73, *P* < 0.0001; *N* = 7–10) (Fig. 2C). Next, we quantified the amount of hTau outside of the pre-synaptic GFP neurons and co-localizing inside of post-synaptic tdTom neurons. We observed no difference in post-synaptic hTau fluorescence between any of the genotypes with all exhibiting very low co-localization signal (main effect of genotype: one-way ANOVA on Tukey-transformed data: *F*(8,71) = 0.78, *P* = 0.62; *N* = 7–10) (Fig. 2D).

### Absence of hTau propagation to post-synaptic neurons in *Drosophila* expressing mutant hTau isoforms combined with global hTau knock-in

**Figure 3:**
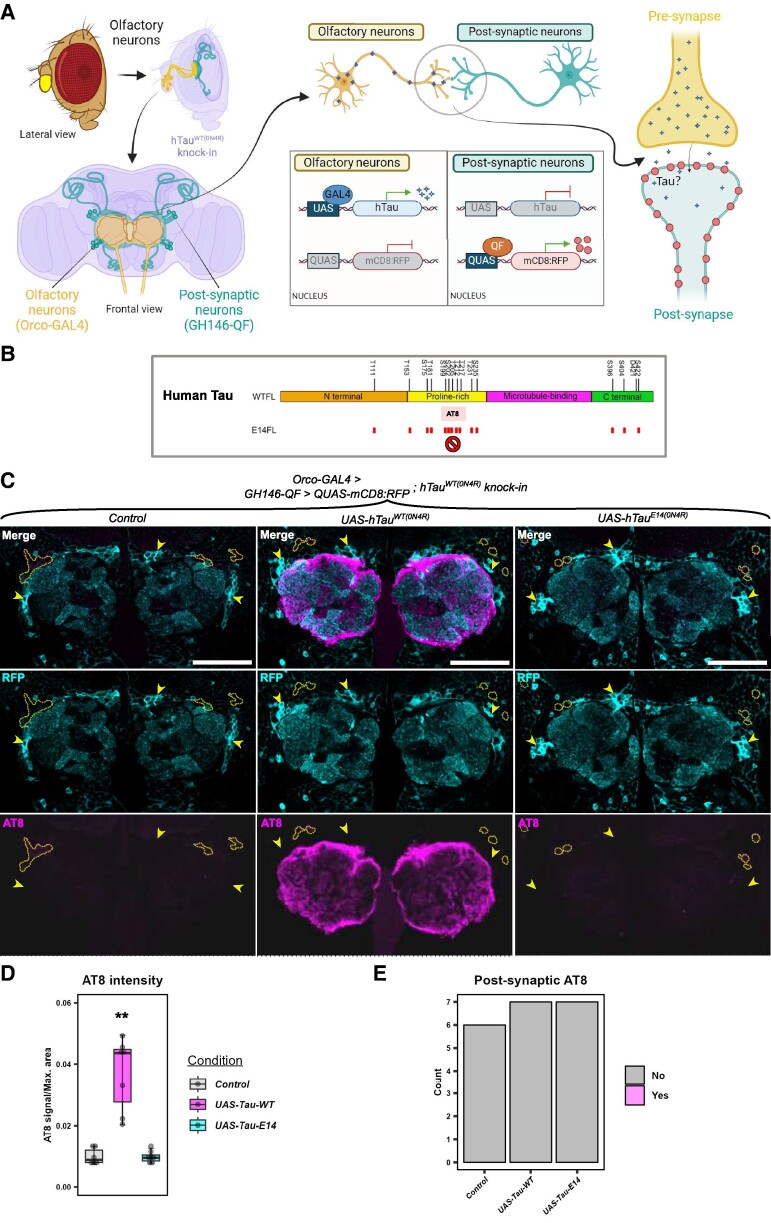
**Absence of hTau propagation in post-synaptic neurons.** (**A**) Schematic shows the brain regions involved in the experiment. Pre-synaptic olfactory neurons are labelled by Orco-GAL4, while PNs connect the antennal lobes to the mushroom body, labelled by GH146-QF. These flies contain a global hTau^WT(0N4R)^ knock-in mutation. Created using BioRender. (**B**) Schematic of hTau adapted from Chi *et al.*^57^ Small bars indicate the locations of the 14 phospho-epitope changes of hTau^E14^. AT8 cannot detect hTau^E14^ due to these mutations. (**C**) In a global hTau^WT(0N4R)^ heterozygous background, GH146-QF labels post-synaptic neurons with membrane-bound red fluorescent protein (mCD8:RFP) while pre-synaptic Orco-GAL4 expresses hTau^WT(0N4R)^ or control. AT8 did not detectably spread trans-synaptically. Arrowheads and dashed areas label clusters of post-synaptic cell bodies and endogenous hTau accumulation, respectively. (**D**) Fluorescence intensity of AT8 was measured and normalized to the maximum area of each brain. AT8 detected significant hTau^WT^ overexpression in the antennal lobes compared to control and hTau^E14^ flies (this difference is indicated by ** above the hTau^WT^ condition, Kruskal–Wallis rank sum test, *χ*^2^ = 13.01, df = 2, *P* = 0.0015; *N* = 6–7 brains), but there was no difference in AT8 staining between control and hTau^E14^ flies. (**E**) AT8 staining within RFP-labelled post-synaptic neurons was not observed. Images were blinded and scored, and no difference between genotypes was observed (*χ*^2^ test, *χ*^2^ = 0.1, df = 2, *P* = 0.95; *N* = 6–7 brains). Scale bar is 50 μm. Genotypes: *Orco-GAL4*; *GH146-QF > QUAS-mCD8:RFP*; *hTau^WT(0N4R)^, Orco-GAL4 > UAS-hTau^WT(0N4R)^*; *GH146-QF > QUAS-mCD8:RFP*; *hTau^WT(0N4R)^, Orco-GAL4 > UAS-hTau^E^*^14^*^(0N4R)^*; *GH146-QF > QUAS-mCD8:RFP*; *hTau^WT(0N4R)^*.

It was possible that 16 PDF neurons were too small a population to lead to overt hTau spread so we decided to use the Orco-GAL4 driver to express hTau in ∼1300^56^ olfactory neurons. As with PDF neurons, olfactory neurons also have their cell bodies distal to their axon termini—in this case, cell bodies are in the antennae and their axons project to the antennal lobes (Fig. 3A). We hypothesized that overexpression of hTau could lead to its spread from pre-synaptic olfactory receptor neurons (ORNs) to post-synaptic projection neurons (PNs). Coupled with this, we reasoned that hTau may be required to be present in post-synaptic neurons to be ‘seeded’ in a prion-like manner by trans-synaptic hTau. Thus, we generated Orco-GAL4; hTau^WT(0N4R)^ and Orco-GAL4; hTau^WT(2N4R)^ knock-in recombinant flies that have endogenous dTau replaced with hTau (Fig. 3A). Two different hTau overexpression transgenes were tested—wild-type UAS-hTau^WT(2N4R)^ and mutant UAS-hTau^P301L(2N4R)^. We stained for AT8 (detects tau phosphorylated at Ser202/Thr205) as we reasoned that staining for total hTau would not be informative due to its abundance in the hTau knock-in. However, we found selective AT8 staining in the hTau^WT(2N4R)^ knock-in controls without hTau overexpression in ORNs (Supplementary Fig. 3A). AT8 staining was observed in a small population of neurons dorsolateral to the antennal lobes. We scored the images for the presence of these AT8-stained neurons and found no difference between genotypes (Supplementary Fig. 3B, Fisher’s exact, *P* = 1). Thus, tau phosphorylated at Ser202/Thr205 appeared to naturally accumulate in this neuronal subset, so it was necessary to be able to identify post-synaptic PNs to detect potential hTau spread. To make sure we could correctly identify PNs, we combined Orco-GAL4; hTau^WT^ recombinants with GH146-QF > QUAS-mCD8:RFP recombinants, which drive RFP expression in PNs. For this experiment, we used the GAL4/UAS and QF/QUAS binary expression systems because the combination of all the required transgenes could not be easily combined with trans-Tango. Our Orco-GAL4/GH146-QF > QUAS-mCD8:RFP; hTau^WT^ recombinants were crossed with control, and two different hTau overexpression transgenes—UAS-hTau^WT(0N4R)^:HA and UAS-hTau^E14(0N4R)^:HA. Phosphomimetic hTau^E14^ carries 14 mutations that render it more toxic than hTau^WT^ and render it invisible to AT8 antibody, which detects phosphorylation at two of the mutated residues (Ser202, Thr205) (Fig. 3B). Thus, we aimed to detect trans-synaptic hTau spread from ORNs to RFP-labelled PNs in a global hTau knock-in background. As before, AT8 stained neurons dorsolateral to the antennal lobes, but these were not overlapping with post-synaptic PNs (see dashed yellow areas in Fig. 3C). We observed robust AT8 staining in ORNs from flies overexpressing hTau^WT(0N4R)^ (Kruskal–Wallis rank sum test, *χ*^2^ = 13.01, df = 2, *P* = 0.0015, Fig. 3D), but AT8 did not capture trans-synaptic hTau spread (*χ*^2^ test, *χ*^2^ = 0.1, df = 2, *P* = 0.95, Fig. 3E).

## Discussion

In this work, we sought to generate a *Drosophila* model of trans-synaptic tau spread. Despite testing multiple different experimental paradigms, we found that *Drosophila* are remarkably resistant to the trans-synaptic tau spread evidenced in human tauopathies and observed in mammalian model systems.^3-12,16,17,21,22,58,59^ These important negative data demonstrate that, while exceptionally useful for a number of other studies in neurodegeneration, including modifiers of tau toxicity,^60-63^  *Drosophila* are unlikely to be a suitable model for screening modifiers of trans-synaptic tau spread. In addition, the resistance to spread seen in this model, compared to mammalian systems, opens up future possibilities to determine factors missing from *Drosophila*, such as proteins, cell types or neuronal functionality^64,65^ that could provide insight into mammalian mechanisms. This also contrasts with fly models successfully showing spread and seeding of toxic neuropathological proteins such as huntingtin,^66-68^ FUS,^69^ TDP-43,^70^ C9orf72 dipeptide repeats^71^ and Aβ.^42,72^

In this study, we perform a robust assessment of suitable tau antibodies for immunostaining experiments. Crucially, we found that the commonly used hTau antibodies, Tau13^73^ and Tau Goat,^22^ showed significant non-specific staining, even in *Drosophila* lacking hTau. This suggests that both antibodies recognize additional non-tau epitopes in *Drosophila* and should be avoided where possible. In this study, we find that using either Tau 5A6 as a full-length tau antibody or AT8 to detect phosphorylated tau provided optimal, specific staining.

In our work, we studied the effect of multiple types of hTau to maximize the likelihood of observing trans-synaptic tau spread. When wild-type 0N4R hTau was overexpressed in PDF neurons, we did not find evidence of hTau spreading beyond the confines of PDF neurons in up to 4-week-old flies. Interestingly, despite tau phosphorylation appearing to be an important process in permitting tau spread in human disease and mammalian models,^74,75^ we also did not find any evidence of tau spread in flies expressing phosphomimetic (E14) 0N4R hTau. Nor did we find evidence of tau spread in flies expressing mutant P301L 2N4R hTau. To further push the system, we tested the hypothesis that hTau may require an hTau template in the post-synaptic compartment in order to ‘seed’ into the post synapse. While studies in mice have found that hTau can spread without mouse tau present in the post-synaptic compartment,^40^ hTau may be required to observe effective tau seeding.^58,76^ We replaced dTau with a wild-type hTau knock-in in all neuronal cells, but still did not detect movement of phosphorylated tau from the pre-synaptic neurons into post-synaptic structures. Taken together, we find that regardless of tau length, phosphorylation status, mutation status or combination with hTau at the post-synapse, *Drosophila* do not show significant trans-synaptic tau spread.

A key advantage of model systems like *Drosophila* is the ability to finely control the experimental environment. Previous studies have shown that raising flies under higher temperatures can exacerbate tau toxicity,^55^ so we performed follow-up experiments in this ‘high-stress’ environment. Interestingly, increasing the temperature had no impact on the detection of wild-type 0N4R hTau or E14 0N4R hTau outside of the PDF neurons.

Given studies in mice and humans have indicated that Aβ can enhance levels of tau spread and toxicity,^38,39^ we examined whether the addition of Aβ to our *Drosophila* model could be sufficient to promote tau spread. Once again, we found that regardless of the length of wild-type hTau expressed in PDF neurons (0N4R compared with 2N4R), or the expression of either human Aβ_40_ or Aβ_42_ from post-synaptic compartments, we could not detect hTau outside of the PDF neurons. This suggests that, in *Drosophila*, combining the two key pathogenic hallmarks of Alzheimer’s disease is not sufficient to induce tau spread, although evidence from other studies shows you can still gain useful insights on the impacts of Aβ and tau on neuronal toxicity and synaptic function.^62,63^ Indeed, in Fig. 2C, post-synaptic Aβ expression does appear to lower hTau expression in pre-synaptic neurons compared to conditions without Aβ, which suggests post-synaptic Aβ toxicity is leading to reduced pre-synaptic hTau levels. *Post hoc* analysis of this data indicates complexity of the Aβ interaction with different hTau isoforms (e.g. Aβ_40_ affects hTau^0N4R^ but not hTau^2N4R^ expression, while Aβ_42_ affects hTau^2N4R^ but not hTau^0N4R^ expression), which may warrant further investigation.

The majority of work in this study was performed using the well-characterized PDF neurons as the primary site for overexpressing tau. PDF neurons are a set of ∼16 neurons known to play key roles in maintaining circadian rhythms.^51^ PDF neurons were chosen as they have axon termini located distally from their cell bodies, allowing us to observe pre-synaptic structures, and their immediate post-synaptic environment, in isolation from the rest of the cell. This permits for more effective assessment of whether tau signals co-localize within the PDF neuron, or whether exogenous tau signal represents genuine spread to post-synaptic compartments. As we did not see tau spread from PDF neurons, we sought to establish whether this is a quirk of this specific neuronal subtype or a consequence of it being a small neuronal population. We performed additional experiments expressing hTau in olfactory neurons, comprising of around 1300 individual cells with axons projecting distal to their cell bodies.^56^ In our hands, we did not observe phosphorylated tau in post-synaptic PNs. Taken together, we show that in two well-characterized neuronal populations in *Drosophila*, overexpression of hTau does not lead to spread of tau to post-synaptic compartments. While we cannot rule out other neuronal circuits being amenable to tau spread, inability to study distal axonal projections will introduce significant noise while imaging, rendering them unsuitable for screening studies. Previous studies have examined the *Drosophila* eye for evidence of tau spread and have shown that tau deposits appear to move from the retina and lamina into the optic lobe during aging, with the distance migrated reduced when tau phosphorylation is lowered.^34,36^ It is unclear, however, whether the migration of tau reported in these papers is a consequence of genuine trans-synaptic spread (as these papers use non-neuronal drivers), or whether tau released from dying photoreceptors has been taken up by other cells.

This study is limited by the use of female flies only. While our choice aligned with established research on *Drosophila* models of neurodegenerative protein propagation^71,77^ and the higher prevalence of Alzheimer’s disease in females, it prevents a comprehensive understanding of potential sex-based differences in hTau spread. Future studies should investigate hTau propagation in both male and female flies to provide a more complete picture of this pathology.

Our inability to detect hTau propagation in *Drosophila* could stem from technical or biological reasons. Technically, the chosen tools might not be ideal for inducing or observing hTau spread in flies. Perhaps, the specific neuronal populations (PDF and Orco) used are naturally resistant, while others might be susceptible. An exhaustive search of all populations was deemed impractical for this study. Detection sensitivity could also be a factor. A minuscule amount of hTau spread might be occurring but remain undetectable with our current methods. Proximity ligation assays, super-resolution imaging or electron microscopy techniques might reveal what standard methods missed. Additionally, our lab’s prior work successfully detected extracellular Aβ using the same microscopy and immunolabelling techniques employed here. This suggests that technical limitations likely are not the only factor preventing hTau spread detection.^42^

Biologically, the mechanisms behind hTau spread in mammals remain unclear. Proposed routes (among others) include passive release from damaged neurons, exosomes, tunnelling nanotubes, and receptor-mediated transmission (as reviewed in Mudher *et al.*,^59^ Goedert *et al.*^78^ and Wei *et al.*^79^). It is possible that either *Drosophila* lack the proteins facilitating human-like hTau spread or their equivalent proteins have different functions or binding partners in flies. Regardless of the cause, the lack of observed hTau spread hinders the usefulness of *Drosophila* as a screening model for overt phenotypes.

Our study prioritized hTau propagation due to its established presence in multiple models and its closer relevance to human disease. However, the potential spread of fly tau (dTau) remains unexplored. Investigating dTau spread could be informative, as it might reveal if the lack of hTau spread is due to the tau type itself or factors within the fly model. For example, overexpressing dTau in Orco neurons within a dTau^−/−^-null background, followed by staining for dTau outside these neurons, could provide valuable insights.

Overall, we conclude that *Drosophila* are highly resistant to trans-synaptic spread of tau in two major central nervous system neuronal populations. This model, while highly useful for studies of toxicity,^62,63^ may therefore not be appropriate to conduct high-throughput screens looking for modifiers of tau spread. Examining why *Drosophila* do not show tau spread as seen in other model systems may provide mechanistic insight that could inform therapeutic development.

## Supplementary Material

fcae256_Supplementary_Data

## Data Availability

All relevant data can be found within the article and its supplementary information and will be available in Edinburgh University Data Repository.
