# Supplementary material for: Drosophila appear resistant to trans-synaptic tau propagation
Source: Brain Commun. 2024 Aug 8;6(4):fcae256. doi: 10.1093/braincomms/fcae256 (PMC11316205; doi:10.1093/braincomms/fcae256)
Supplement: fcae256_Supplementary_Data [file fcae256_supplementary_data.pdf]

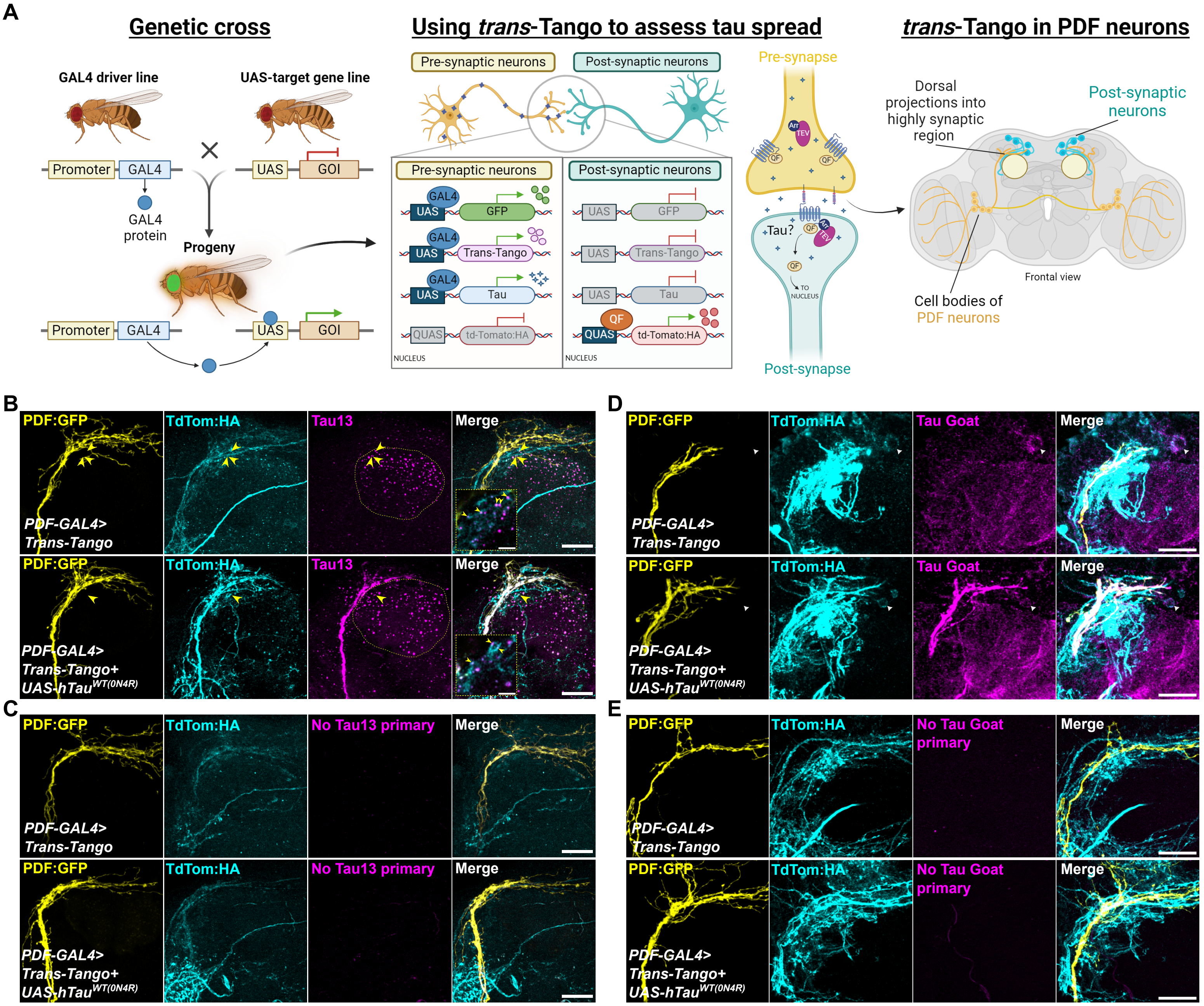

Supplementary Figure 1. Non-specific binding by human tau antibodies - Tau13 and Tau Goat - in *Drosophila* brains. (**A**) The trans-Tango system in *Drosophila* utilizes a synthetic signalling pathway to map neural circuits. All neurons express a trans-Tango glucagon receptor (G-protein coupled) and a proteolytic complex that can cleave the trans-Tango receptor (Arr-TEV). Specific presynaptic neurons are genetically labelled using the GAL4/UAS system to drive expression of a tethered ligand (membrane-bound synaptic protein with tethered glucagon) and genes-of-interest (GOI – e.g. GFP, hTau). This ligand binds to the trans-Tango receptor on postsynaptic partners, triggering release of a transcription factor (QF) from the receptor via proteolysis. The reporter, translocates to the nucleus where it binds QUAS elements allowing labelling of postsynaptic neurons (e.g. tdTom). (**B**) The hTau-specific antibody, Tau13, labels specific punctate structures in the calyx of the mushroom body (neuropil outlined with dashed yellow in the Tau13 channel). This staining is observed even in conditions that do not contain hTau expression. Some of these puncta colocalise with post-synaptic dendrites (yellow arrowheads). (**C**) Punctate neuropil staining is absent in conditions incubated without the primary antibody (Tau13). (**D**) Tau Goat labels apparent cell bodies in the Kenyon cell area of the mushroom body. Some of these cell bodies colocalise with post-synaptic cell body staining (white arrowheads). This staining is observed even in conditions that do not contain hTau expression. (**E**) Cell body staining is absent in conditions incubated without the primary antibody (Tau Goat). Scale bar is 20 µm for all micrographs except zoomed insets which is 5µm. Genotypes: *PDF-GAL4>trans-Tango, PDF-GAL4>trans-Tango + UAS-hTau*$^{WT(0N4R)}$.

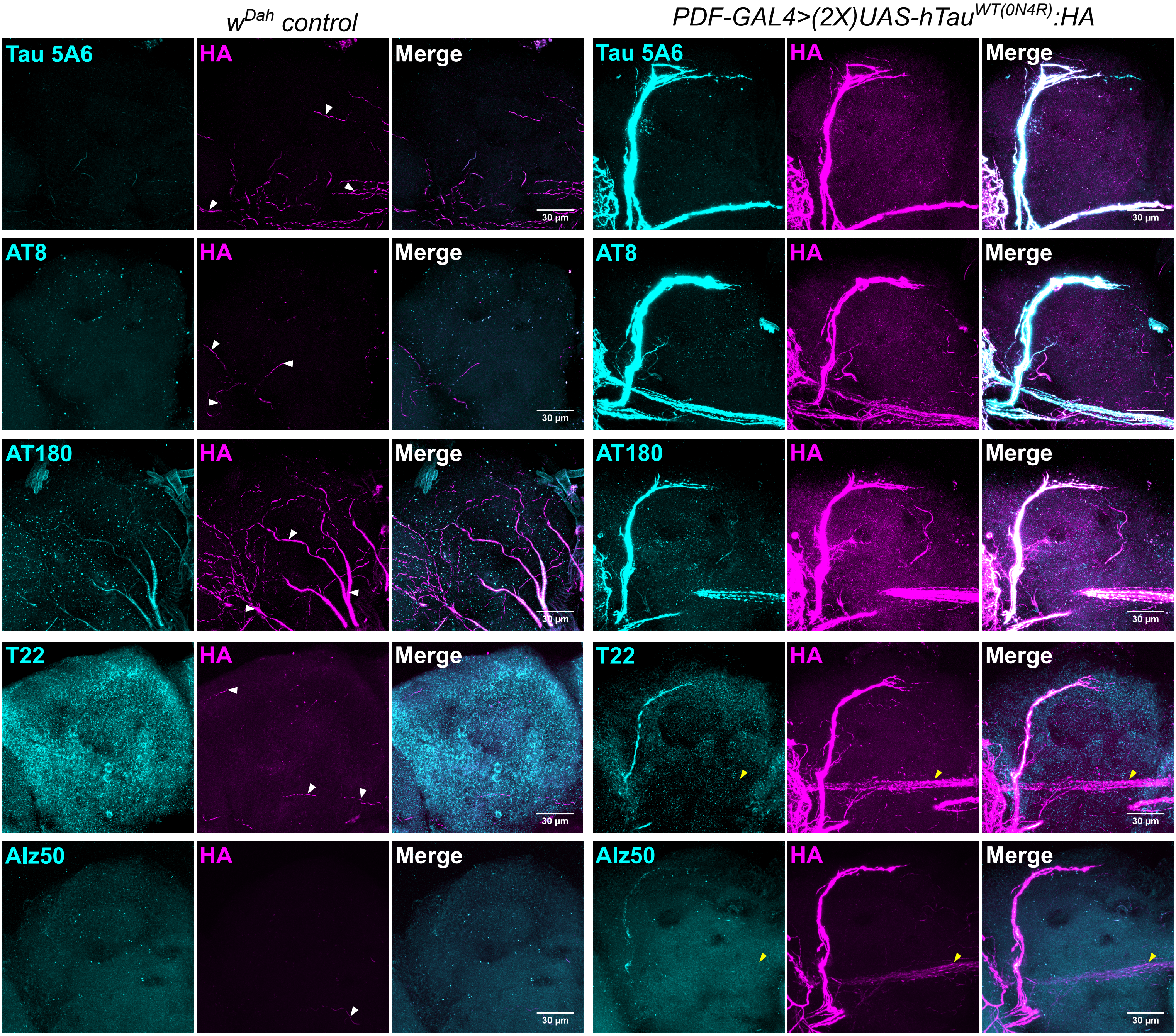

Supplementary Figure 2. Absence of hTau propagation using different hTau antibodies.
Other hTau antibodies could detect HA-tagged hTau expressed in PDF neurons, though the oligomeric (T22) and misfolded (Alz50) forms of hTau had weaker expression and did not fully label all hTau-expressing neurons indicating lower levels. Yellow arrowheads indicate the PDF-expressing posterior optic tract that does not contain staining for oligomeric (T22) or misfolded (Alz50) hTau. Scale bar = 30 µm. White arrowheads indicate autofluorescence from trachea – particularly extensive in the AT180-stained control brain. Genotypes: w^{Dah}, *PDF-GAL4>UAS-hTau*$^{WT(0N4R):HA}$.

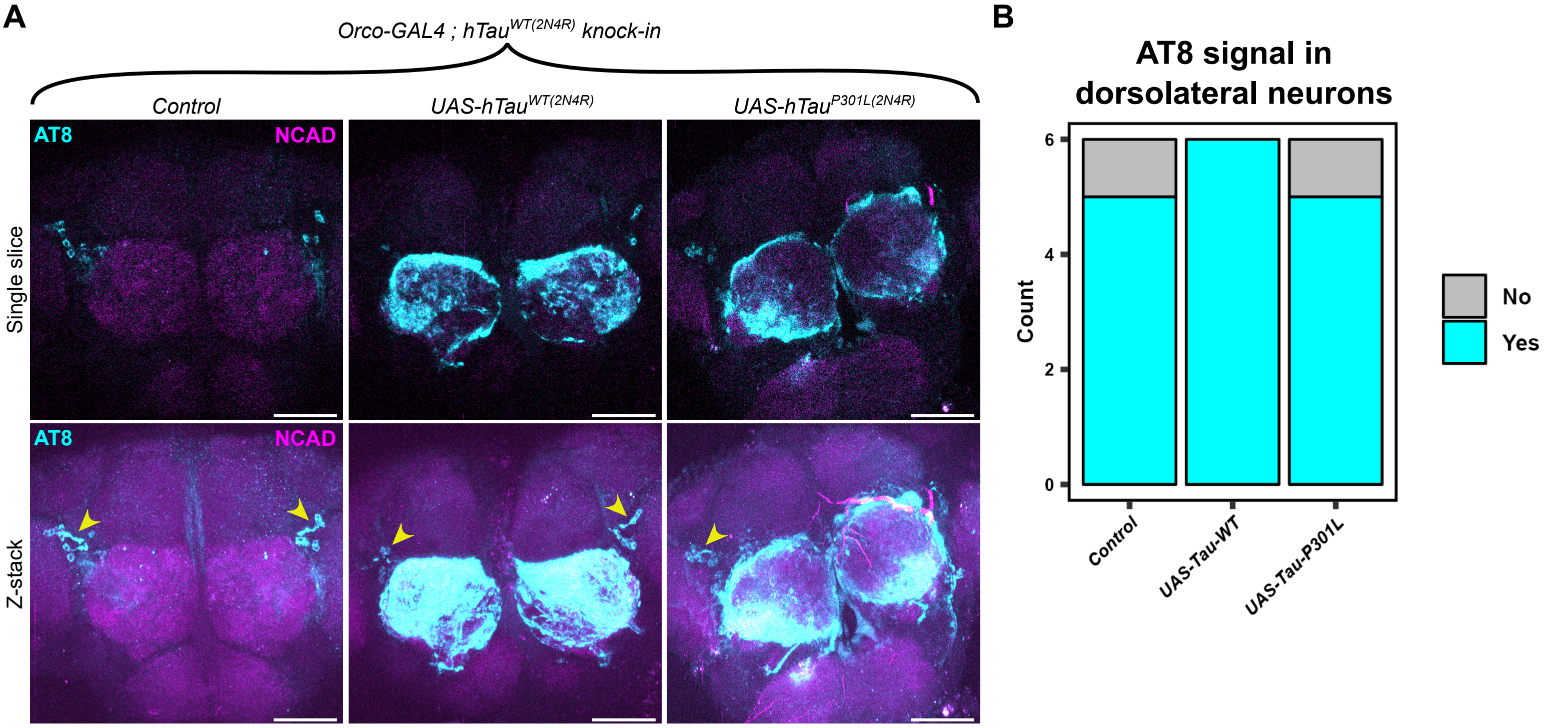

Supplementary Figure 3. AT8 staining in hTau knock-in flies.
(**A**) Single confocal slices and z-stacks from 6-week-old flies heterozygous for hTauWT(2N4R) knock-in. We observed consistent AT8 staining in a small subset of neurons (yellow arrowheads) dorsolaterally to the antennal lobes that was present even in controls without hTau overexpression. (**B**) AT8 staining of the dorsolateral neurons (i.e. not Orco) in the region surrounding the antennal lobes was found in all genotypes. Fisher's exact test, chosen for its suitability with small sample size, detected no significant association between genotype and AT8 signal ($p = 1$; N=6 brains). Scale bar is 50 μm. Genotypes: *Orco-GAL4/+; hTau$^{WT(2N4R)}$, Orco-GAL4>UAS-hTau$^{WT(2N4R)}$; hTau$^{WT(2N4R)}$, Orco-GAL4>UAS-hTau$^{P301L(2N4R)}$; hTau$^{WT(2N4R)}$*.
